# Genomic Insights Into Antimicrobial Resistance and Virulence of *Enterococcus avium* Strains From Bovine Mastitis in Some Selected Dairy Farms of Bangladesh

**DOI:** 10.1002/vms3.71060

**Published:** 2026-06-29

**Authors:** Monira Rahaman, Md. Morshedur Rahman, Kh. Yeashir Arafat, Md. Abu Ahsan Gilman, Naim Siddique, Taniya Sultana, Ziban Chandra Das, Anup Kumar Talukder, Tofazzal Islam, A. N. M. Aminoor Rahman, M. Nazmul Hoque

**Affiliations:** ^1^ Molecular Biology and Bioinformatics Laboratory, Department of Gynaecology Obstetrics and Reproductive Health, Gazipur Agricultural University Gazipur Bangladesh; ^2^ Institute of Biotechnology and Genetic Engineering Gazipur Agricultural University Gazipur Bangladesh

**Keywords:** bovine mastitis, *enterococcus avium*, multidrug resistance, one health, virulence factors

## Abstract

**Background:**

Bovine mastitis remains a major global threat to dairy production and animal welfare, with the increasing emergence of multidrug‐resistant (MDR) pathogens severely undermining the efficacy of conventional antimicrobial therapies and complicating disease control strategies. *Enterococcus avium*, traditionally understudied in livestock, has been occasionally associated with mastitis and poses potential zoonotic and antimicrobial resistance (AMR) risks.

**Objectives:**

This study aimed to investigate the prevalence, AMR, virulence repertoire and genomic features of *E. avium* isolates from milk, faeces and soil in some selected dairy farms with mastitis in Bangladesh.

**Methods:**

A total of 110 samples (milk, faecal and soil) were collected and screened for *E. avium* using selective culture and polymerase chain reaction (PCR). Antimicrobial susceptibility was determined against 15 antibiotics. Four MDR *E. avium* isolates (4M1, 4S1, 4F1 and 4F2) were selected for whole‐genome sequencing (WGS) to characterize their genomic diversity, functional potential, resistome and virulome in dairy cows and associated environments.

**Results:**

*E. avium* was detected in 56.36% of samples, with highest prevalence in milk (47.8%). MDR was highly prevalent (93.5%), with frequent resistance to sulphonamides, nitrofurantoin and oxacillin, whereas gentamicin retained activity. Genomic analyses revealed conserved core genomes alongside variable accessory elements, indicating both evolutionary stability and adaptive potential. Phylogenetic proximity to human‐derived strains highlights zoonotic risk. Functional profiling demonstrated robust metabolism, environmental sensing, adhesion‐related virulence factors and multiple bacteriocin clusters, supporting persistence and microbial competition. ARGs conferring multidrug efflux, β‐lactam and fluoroquinolone resistance were conserved across isolates.

**Conclusions:**

*E. avium* is a prevalent mastitis pathogen, highly conserved among the studied isolates, with significant virulence and AMR profiles, highlighting its zoonotic potential and need for One Health–based surveillance strategies.

## Graphical abstract

The graphical abstract outlines that samples from milk, faeces and soil were tested for *Enterococcus avium* using culture, PCR and genome sequencing.2. Overall, prevalence of *E. avium* was 56.3%, and 93.5% of isolates were multidrug‐resistant.3. Genomic analysis revealed six ARGs, 43–45 VFGs and 4–6 bacteriocins.4. A human isolate shared 100% identity with a study strain from dairy sources.5. Conserved genetics among milk, faeces, soil and human isolates highlight zoonotic AMR risk.

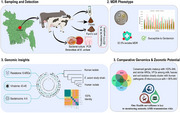

## 1 Introduction

Bovine mastitis remains one of the most prevalent and costly diseases affecting the global dairy industry, with substantial economic losses attributed to decreased milk yield, altered milk quality and treatment expenses (Hoque et al. 2019, 2020). Traditionally, mastitis has been associated with well‐characterized pathogens such as *Staphylococcus aureus*, *Escherichia coli* and *Streptococcus uberis* (Ashraf and Imran 2020). However, emerging and opportunistic microorganisms are increasingly implicated in intramammary infections, complicating diagnosis, treatment and prevention strategies (Nam et al. 2010; Seker et al. 2023). A major concern in mastitis management is the rising prevalence of antimicrobial resistance (AMR) among pathogens (Hoque et al. 2020; Krömker and Leimbach 2017). Extensive antimicrobial use in intensive livestock systems has promoted the emergence and spread of multidrug‐resistant (MDR) microorganisms, particularly in low‐ and middle‐income countries with limited diagnostics and regulatory oversight. MDR *enterococci* not only compromise animal health but also pose public health risks through zoonotic transmission, environmental contamination and foodborne exposure (Tang et al. 2023; Aslam et al. 2024). Often termed the ‘Silent Pandemic’, AMR is accelerated by misuse in human and veterinary medicine and represents a critical One Health threat, demanding urgent, coordinated action across human, animal and environmental sectors (Founou et al. 2021).

Enterococci are resilient Gram‐positive bacteria that inhabit the gastrointestinal tracts of humans and animals and persist in diverse environmental reservoirs (Byappanahalli et al. 2012). Although species, such as *Enterococcus faecalis* and *Enterococcus faecium*, have been extensively studied due to their role in hospital‐acquired infections and as reservoirs of vancomycin resistance, other species in this genus are increasingly implicated in infections across both human and veterinary domains (Arias and Murray 2012; Butaye et al. 2001; Jiménez et al. 2013; Hammerum 2012). Among these, *Enterococcus avium* is emerging as a clinically relevant species capable of infecting both animals and humans, often exhibiting MDR (Coccitto et al. 2023; Toc et al. 2022). Notably, this bacterium has been isolated from hospitalized patients and linked to serious infections including brain abscesses, intra‐abdominal infections, endocarditis, bacteraemia, peritonitis and intracranial suppurative infections (Mohanty et al. 2005; Chao et al. 2013; Patel et al. 1993; Yu et al. 2019). *E. avium* has occasionally been isolated from milk of mastitic cows and reported as a potential opportunistic pathogen in dairy environments (Nam et al. 2010; Dapkevicius et al. 2021; Guimarães et al. 2024). The presence of *E. avium* in dairy environments presents a dual concern: Its involvement in bovine mastitis and its potential as a zoonotic reservoir for AMR determinants that can disseminate through the food chain, environment or direct animal–human contact (Zaidi et al. 2024; Zaheer et al. 2020). Despite the emerging recognition of *E. avium* as a pathogen in mastitis, studies investigating its AMR spectrum, virulence gene repertoire and adaptive genomic features in livestock settings are scarce. Existing investigations have largely concentrated on enterococci isolated from clinical human sources (Palmer et al. 2014), leaving a significant knowledge gap concerning their evolutionary trajectories, resistance dynamics and health implications in agricultural environments. This gap is particularly critical given the recognized role of enterococci as reservoirs of transferable AMR determinants, which may circulate between animal, human and environmental niches.

In parallel with phenotypic resistance profiling, genomic approaches have become essential for characterizing microbial populations, tracking the spread of resistance genes and identifying molecular determinants of virulence and adaptation (Hoque et al. 2024; Rahman et al. 2024; Sultana et al. 2025). The increasing accessibility of whole‐genome sequencing (WGS) has revolutionized our understanding of bacterial evolution, pathogenicity and AMR. WGS enables high‐resolution analyses of species boundaries, resistance determinants, virulence genes, mobile genetic elements and evolutionary relationships (Rahman et al. 2025). In dairy farming, genomic characterization of mastitis‐associated bacteria is crucial for detecting emerging threats, monitoring AMR dissemination and guiding intervention strategies within a One Health framework. In this study, we address this gap by analysing *E. avium* isolates from milk, faecal and soil samples collected from dairy farms with mastitis‐affected cows in Bangladesh. Using an integrated approach of antimicrobial susceptibility testing, species identification and WGS‐based comparative genomics, we examined genomic diversity, resistome composition, virulence factors, metabolic pathways and bacteriocin gene clusters. This represents the first genomics‐driven investigation of mastitis‐associated *E. avium*, providing insights into its pathogenic potential, ecological adaptability and epidemiological relevance in livestock systems.

## 2 Methodology

### 2.1 Sample Collection and Processing

A total of 110 samples were collected from 45 smallholder dairy farms in the Gazipur district (24.09° N, 90.41° E) of Bangladesh (Mallick et al. 2025). Cows were diagnosed with clinical mastitis (CM) based on observable clinical signs and a California Mastitis Test (CMT) score of 3, which indicates a strong positive reaction with pronounced gel formation (Hoque et al. 2015). Clinical signs included abnormal milk (discolouration or clots), udder swelling, redness and increased local temperature (Hoque et al. 2015, 2024). The collected samples comprised 45 CM milk samples (from 45 lactating cows), 35 faecal samples (selected based on freshness and availability) and 30 farm soil samples (based on availability). Approximately 10 mL of milk and 5g of faeces or soil were collected in sterile falcon tubes during morning milking (08:00–10:00 h), following strict teat‐end disinfection and hygienic procedures (Sultana et al. 2024). The samples were transported on ice (4°C) to the laboratory and processed within 3–5 h. Milk samples were cultured directly, whereas faecal and soil samples were diluted with distilled water at 1:1 ratio, homogenized by vortexing and stored appropriately (at 4°C for 2–3 days) for subsequent analyses.

### 2.2 Isolation and Identification of Bacteria

Milk, homogenized faeces and soil samples (500 µL) were inoculated into 5 mL nutrient broth (HiMedia, India) and incubated overnight at 37°C. The overnight broth culture was plated on nutrient agar (Oxoid, UK) and incubated overnight at 37°C, followed by streaking on Modified Edward Medium (MEM; Oxoid, UK) and incubation for 24–48 h at 37°C. Well‐isolated colonies (small, brownish‐black colonies) were subcultured onto blood agar (Oxoid, UK) supplemented with 5% sheep blood and incubated aerobically at 37°C for 48 h. Pure colonies were obtained by subsequent streaking on MEM agar (HiMedia, India). Phenotypic identification of the isolates was performed by examining colony morphology and Gram staining (Gram‐positive, chain‐forming and purple‐stained colonies), whereas genus‐level identification of *Enterococcus* spp. was attempted through polymerase chain reaction (PCR) using the primers *Ent1* (5′‐TACTGACAAACCATTCATGATG‐3′) and *Ent2* (5′‐AACTTCGTCACCAACGCGAAC‐3′) (Ke et al. 1999). PCR amplification was carried out in a 20 µL reaction mixture containing 50 ng of template DNA, 0.2 µM of each primer, 0.2 mM dNTPs, 1.5 mM MgCl_2_ and 0.4 U Taq DNA polymerase in KCl buffer (Fermentas). Thermal cycling consisted of an initial denaturation at 94°C for 5 min, followed by 40 cycles of 94°C for 30 s, 51°C for 1 min and 72°C for 30 s, with a final extension at 72°C for 5 min. PCR products were resolved on a 1.5% agarose gel in TAE buffer, stained with ethidium bromide and visualized under a UV transilluminator. Finally, VITEK‐2 system v9.01 (Compact system, Mérieux) identified the isolates as *E. avium* (Joyanes et al. 2001; Mallick et al. 2024).

### 2.3 Antimicrobial Susceptibility Testing

The antimicrobial susceptibility of 62 confirmed *E. avium* isolates was determined using the Bauer‐Kirby disk diffusion method (Hoque et al. 2020) following CLSI M100 34rd Edition guidelines (Rai et al. 2023). Fifteen antibiotics from 12 classes were tested, including penicillins (ampicillin 10 µg and oxacillin 1 µg), tetracyclines (doxycycline 30 µg and tetracycline 30 µg), nitrofurans (nitrofurantoin 300 µg), quinolones (ciprofloxacin 10 µg), cephalosporins (cefoxitin 30 µg), penems (imipenem 10 µg), aminoglycosides (gentamicin 10 µg), phenols (chloramphenicol 30 µg) and macrolides (erythromycin 15 µg). In brief, bacterial suspensions from overnight nutrient broth cultures were evenly spread on nutrient agar plates using sterile swabs, rotating the plate to ensure uniform coverage. Antibiotic discs were placed with adequate spacing to prevent overlapping zones, and plates were incubated at 37°C for 18–24 h (Hoque et al. 2024). After incubation, the diameter of inhibition zones around each disc was measured with a ruler, and susceptibility was interpreted according to CLSI 2023 guidelines. Isolates resistant to three or more antibiotic classes were classified as MDR.

### 2.4 Genome Sequencing, Assembly and Annotations

Four MDR *E. avium* isolates (4M1, 4F1, 4F2 and 4S1), each resistant to more than eight antibiotics, were selected for WGS. Genomic DNA was extracted using the QIAamp DNA Mini Kit (QIAGEN, Hilden, Germany) after overnight cultivation in nutrient broth (Biolife, Italy) at 37°C. Sequencing libraries were prepared from 1 ng of DNA with the Nextera DNA Flex Kit (Illumina, USA) and sequenced on an Illumina MiSeq platform using a 2 × 250 bp paired‐end protocol. Raw reads were quality filtered with Trimmomatic v0.39 (Bolger et al. 2014) and assessed with FastQC v0.11.7 (Andrews et al. 2010). Reads with Phred scores above 20 were assembled de novo using SPAdes v3.15.5 (Bankevich et al. 2012). Assembled genomes were annotated using both the NCBI Prokaryotic Genome Annotation Pipeline (PGAP v6.6) (Tatusova et al. 2016) and the RAST v2.0 server (Overbeek et al. 2014). Genome completeness and quality were evaluated with CheckM v1.2.2 (Parks et al. 2015) using the *Enterococcus*‐specific marker set. Functional categorization and visualization were performed in Genovi v0.2.16 (Cumsille et al. 2023), with predicted genes assigned to Clusters of Orthologous Groups (COG) (Galperin et al. 2025) functional classes and summarized in bar charts. Further, all the genomes were annotated using the KEGG database (Kanehisa et al. 2016) with the KEGG Automatic Annotation Server (KAAS)https://www.genome.jp/kegg/kaas/ for their metabolic pathway analysis.

### 2.5 Phylogenetic and Pangenome Analysis

Phylogenetic relationships were inferred from *16S rRNA* gene sequences of the four *E. avium* genomes (4M1, 4F1, 4F2 and 4S1), together with 29 publicly available *Enterococcus* reference genomes (Table S1) retrieved from NCBI. The *16S rRNA* genes were extracted using Barrnap v0.9 (Seemann 2013) on the Galaxy platform. A maximum‐likelihood phylogenetic tree was generated with MEGA11 (Tamura et al. 2021) and visualized using the Interactive Tree of Life (iTOL v6) (Letunic and Bork 2024). On the basis of phylogenetic clustering, the four study genomes and their five most closely related *Enterococcus* reference genomes were further analysed for average nucleotide identity (ANI) using ANIclustermap (Musiał et al. 2024). Further, PPanGGOLiN (Gautreau et al. 2020) was used for pangenome analysis to investigate their genomic diversity.

### 2.6 Bacteriocin Gene Cluster Analysis in *E. avium* Strains

Bacteriocin gene clusters in all four sequenced genomes were identified using the BAGEL v.4.0 (van Heel et al. 2018) web tool. Additionally, the predicted bacteriocin domains were manually verified through BLASTp (Lavigne et al. 2008) search against non‐redundant protein sequences (nr) database.

### 2.7 Analysis of Resistance and Virulence Determinants in *E. avium* Strains

For the detection of ARGs, the four *E. avium* genomes (4M1, 4F1, 4F2 and 4S1) were annotated with RAST server, NCBI PGAP and comprehensive antibiotic resistance database (CARD v3.2.4) (Jia et al. 2017). We comprehensively analysed the assembled dataset using PlasmidFinder v2.0.1 (Carattoli and Hasman 2019) to identify plasmid replicons and known plasmid‐associated resistance genes. Further, the Virulence Factor Database (VFDB v6.0) (Chen et al. 2016) used for the predicted virulence factor genes (VFGs).

### 2.8 Statistical Analysis

Data were entered into Microsoft Excel 2016 (Microsoft Corp., Redmond, WA, USA) and analysed using Excel. The AMR patterns, resistant, intermediate resistant and sensitivity were calculated using the CLSI 2023 guideline using the cut‐off as provided in the brochure of the manufacturer (Oxoid, Hampshire, UK).

## 3 Results

### 3.1 Phenotypic Characterization of *E. avium* Isolates

**FIGURE 1:**
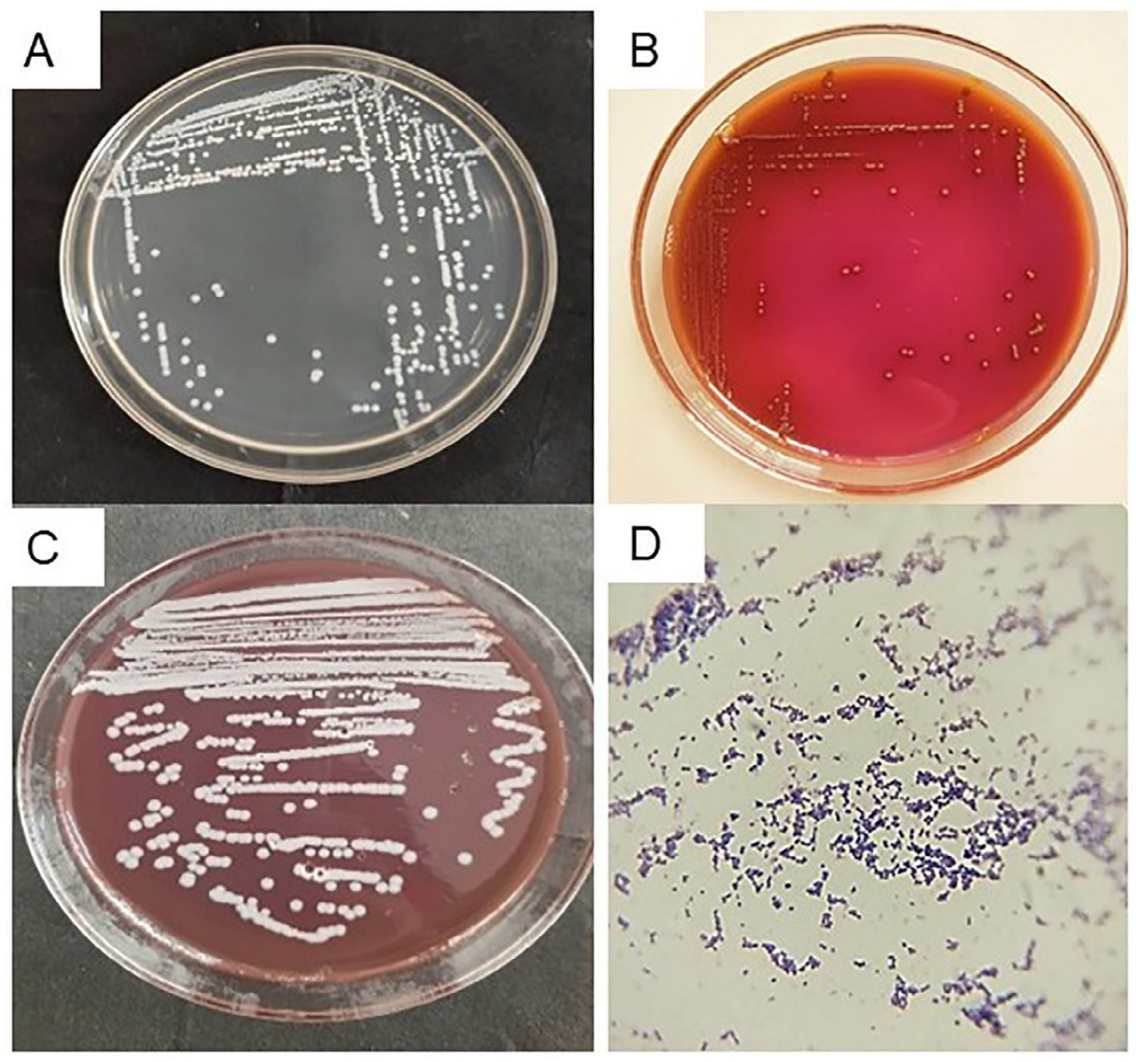
Morphological characteristics of *Enterococcus avium* isolates on different culture media and Gram staining. (A) Circular, white, smooth and translucent colonies on nutrient agar. (B) Colourless colonies observed on Modified Edward Medium (MEM) agar. (C) Large, single, smooth and whitish colonies on blood agar, showing typical *E. avium* growth. (D) Gram‐stained cells of *E. avium* showing Gram‐positive, purple‐coloured cocci arranged in chains under 100× magnification.

The *E. avium* isolates exhibited distinct morphological characteristics depending on the culture medium. On nutrient agar, colonies were circular, white, smooth and translucent (Figure 1A). On MEM agar, the colonies appeared colourless (Figure 1B), whereas on blood agar, the isolates formed large, single, smooth and whitish colonies, representing typical growth of *E. avium* (Figure 1C). Gram staining confirmed the identity of the isolates, with all suspected *E. avium* cells appearing as Gram‐positive, purple‐coloured cocci arranged in chains under light microscopy at 100× magnification (Figure 1D).

### 3.2 Prevalence of *E. avium* Associated Mastitis

**FIGURE 2:**
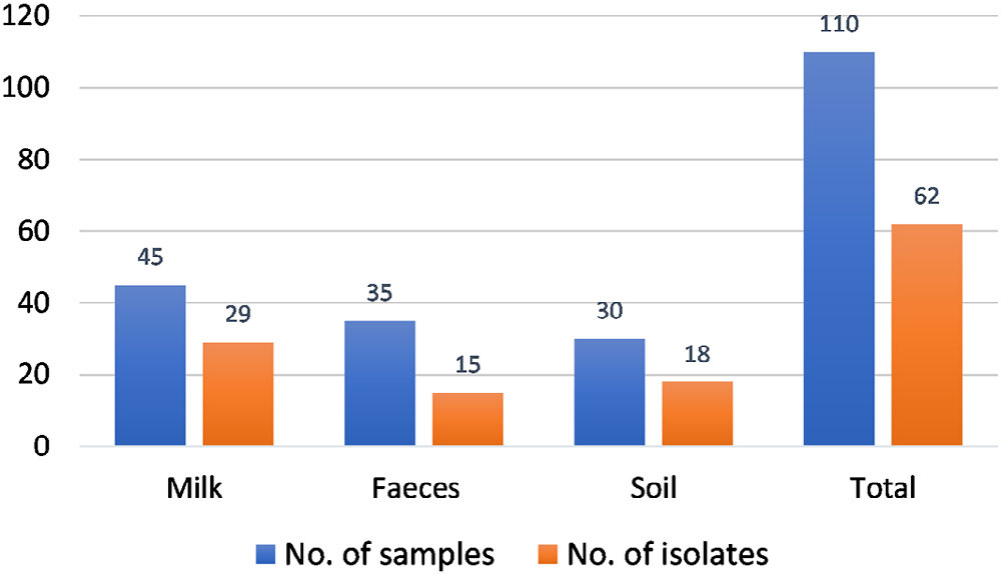
Prevalence of *Enterococcus avium* isolates across mastitic milk, faeces and farm soil.

A total of 62 out of 110 samples (56.36%) were confirmed as *E. avium* using selective culture methods, and source‐specific analysis revealed that milk samples accounted for 29 isolates (47.77%), faecal samples for 15 isolates (29.03%) and soil samples for 18 isolates (24.19%) (Figure 2).

### 3.3 Antibiogram Profile of *E. avium* Isolates

**FIGURE 3:**
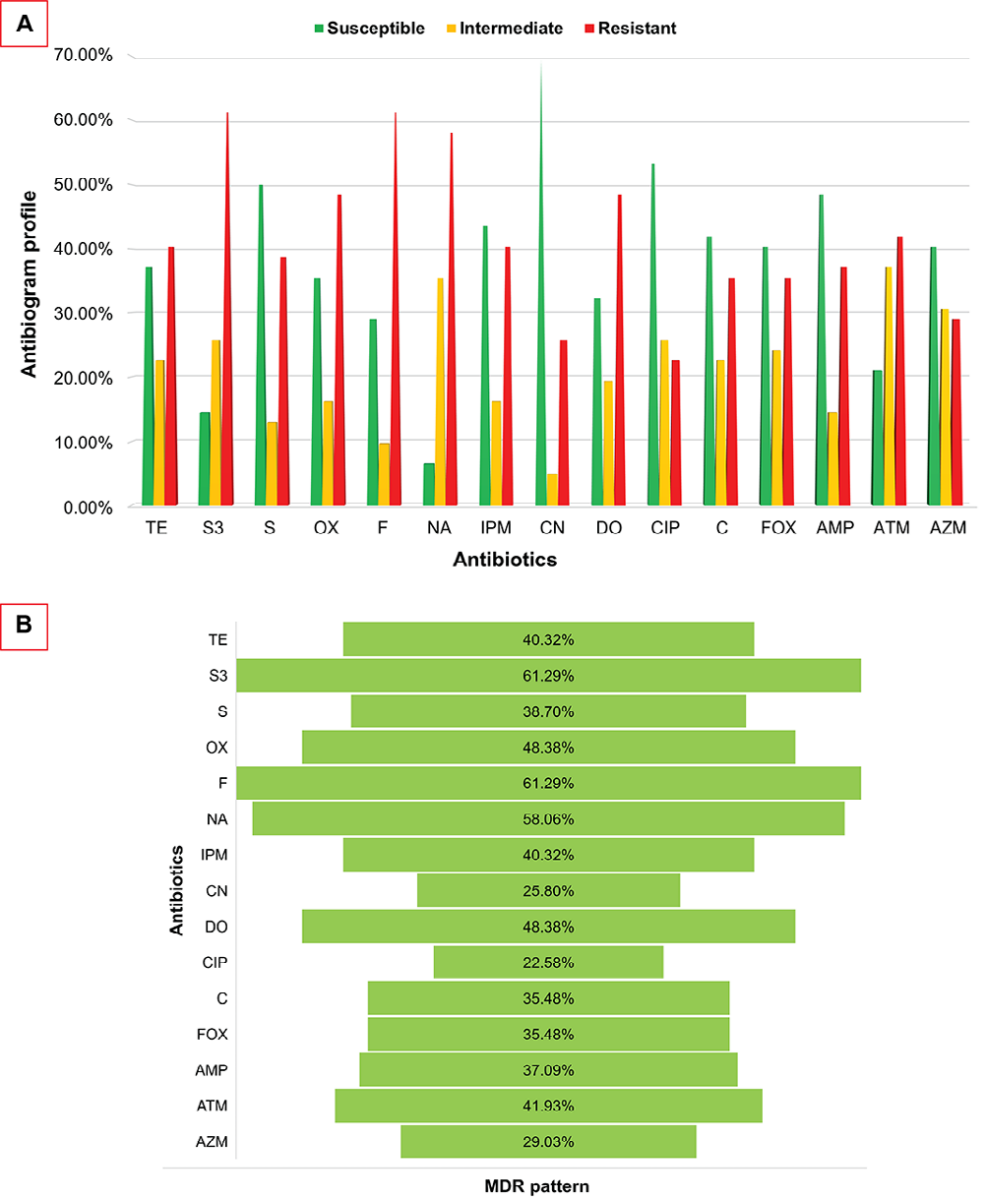
Antimicrobial resistance and multidrug resistance patterns of *Enterococcus avium* isolates (*n* = 62). The overall antibiogram profile (A) shows varying resistance levels across tested antibiotics, whereas panel (B) illustrates the multidrug resistance (MDR) patterns observed. Antibiotics tested include AZM (azithromycin), ATM (aztreonam), AMP (ampicillin), FOX (cefoxitin), C (chloramphenicol), CIP (ciprofloxacin), DO (doxycycline), CN (gentamicin), IPM (imipenem), NA (nalidixic acid), F (nitrofurantoin), OX (oxacillin), S (streptomycin), S3 (sulphonamides) and TE (tetracycline).

The in vitro antibiogram analysis of 62 *E. avium* isolates against 15 antibiotics from 12 different classes revealed distinct resistance and susceptibility patterns (Figure 3). High resistance profiles were observed against nitrofurantoin and sulphonamides (60%–70%), followed by nalidixic acid (50%–60%) and oxacillin, doxycycline, tetracycline, imipenem, azithromycin and aztreonam (40%–50%). Moderate resistance (30%–40%) was recorded for streptomycin, cefoxitin and chloramphenicol (Figure 3A). Conversely, the isolates exhibited notable susceptibility to gentamicin (60%–70%), indicating its potential efficacy against *E. avium*. Moderate susceptibility was also observed for ciprofloxacin and streptomycin (50%–60%), whereas ampicillin and imipenem showed comparatively lower activity (40%–50%). Markedly lower susceptibility was recorded for sulphonamides (14.51%) and nalidixic acid (6.45%), reflecting limited therapeutic effectiveness of these drugs. MDR was highly prevalent, with 93.54% of isolates resistant to ≥3 antibiotic classes. A subset of isolates displayed extreme resistance, with resistance to as many as 11 antibiotics (Figure 3B). Among these MDR isolates, the highest resistance frequencies were against nitrofurantoin (61.29%), sulphonamides (61.29%) and nalidixic acid (58.06%) (Figure 3B).

### 3.4 Genomic Feature of *E. avium* Strains

**FIGURE 4:**
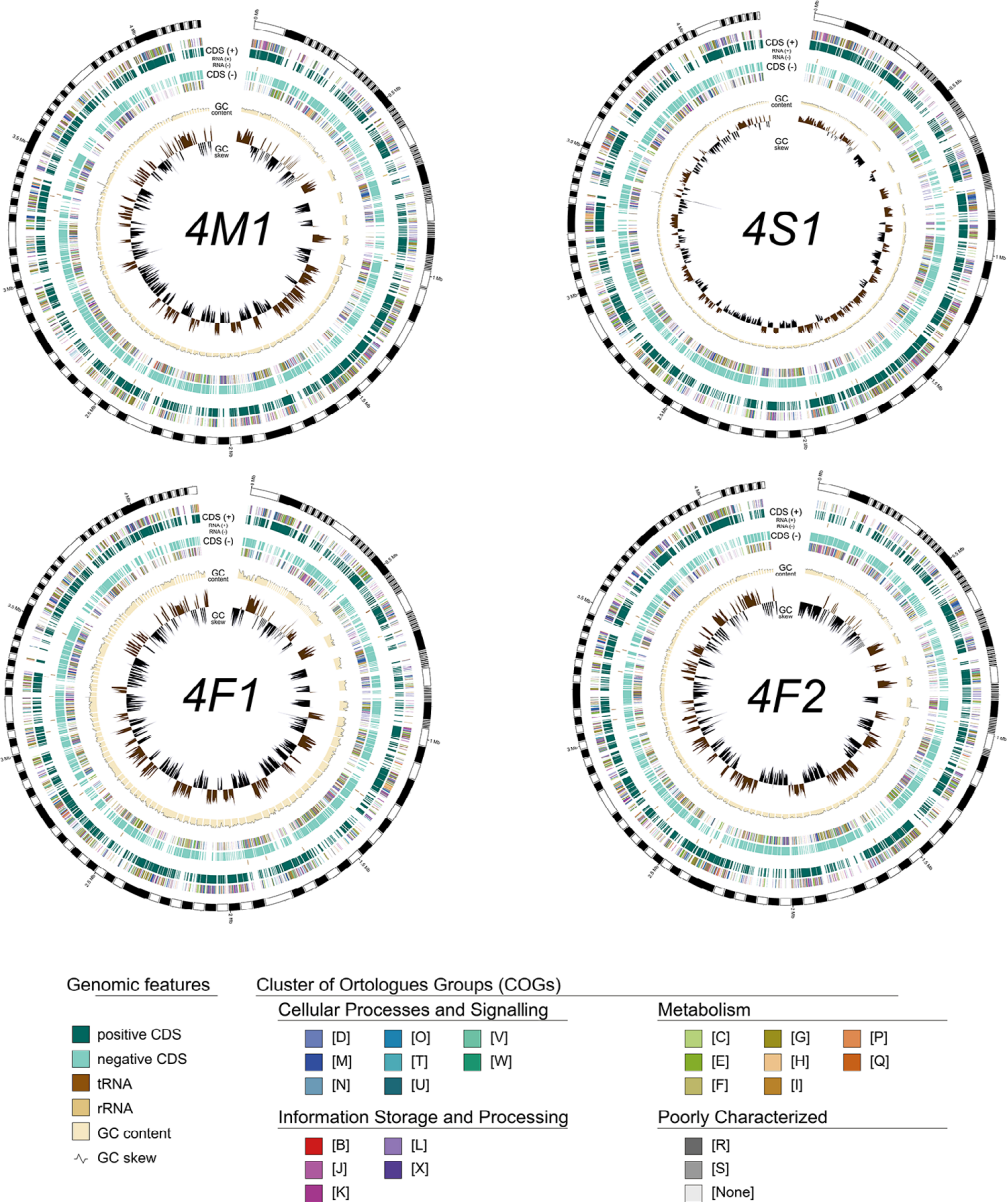
Circular genome maps of *Enterococcus avium* strains 4M1, 4S1, 4F1 and 4F2. Each map displays genomic features from the outermost to the innermost ring: genome contigs, coding sequences (CDS) on the forward strand annotated with COG categories, tRNA and rRNA locations, CDS on the reverse strand with COG annotation, GC content and GC skew.

Four MDR *E. avium* strains, including one from milk (4M1), two from faeces (4F1 and 4F2) and one from soil (4S1), were selected for WGS (Table 1). All genomes were approximately 4.2 Mb in size, exhibited a GC content of 39.1%, and were sequenced to an average coverage of 66.5×. The predicted coding potential was largely conserved across the isolates, with 4131, 4130, 4132 and 4173 coding sequences (CDSs) identified in 4M1, 4F1, 4F2 and 4S1, respectively. The number of RNA genes varied slightly, ranging from 55 in faecal isolates 4F1 and 4F2 to 57 in 4M1 and 74 in the soil isolate 4S1. Detailed genomic characteristics are presented in Table 1, whereas circular genome maps of the isolates are shown in Figure 4. Furthermore 256 sub‐system features annotated in 4M1 and 4S1 genomes, where 255 sub‐system features in both genomes (4F1 and 4F2) isolated from faecal sample using RAST server.

**TABLE 1:** General genomic features of the *Enterococcus avium* strains.

Features	*E. avium* strains			
	4M1	4F1	4F2	4S1
Genome size (bp)	4,213,172	4,215,104	4,212,140	4,246,665
Genome coverage	66.5×	66.5×	66.5×	66.5×
GC content (%)	39.1	39.1	39.1	39.1
Total contigs	170	165	173	155
Contig *N*_50_ (bp)	44,465	45,550	43,365	51,547
*L*_50_	29	28	30	25
Total genes	4188	4185	4187	4247
CDSs	4131	4130	4132	4173
Protein coding genes	4083	4082	4086	4126
RNA genes	57	55	55	74
tRNA genes	48	46	46	60
rRNAs	1, 3, 1 (5S, 16S, 23S)	1, 3, 1 (5S, 16S, 23S)	1, 3, 1 (5S, 16S, 23S)	3, 4, 3 (5S, 16S, 23S)
ncRNAs	4	4	4	4
Pseudo genes	48	48	46	47
No. of subsystems	256	255	255	256

Abbreviation: CDS, coding sequences.

### 3.5 Evolutionary Analysis of *E. avium* Strains

**FIGURE 5:**
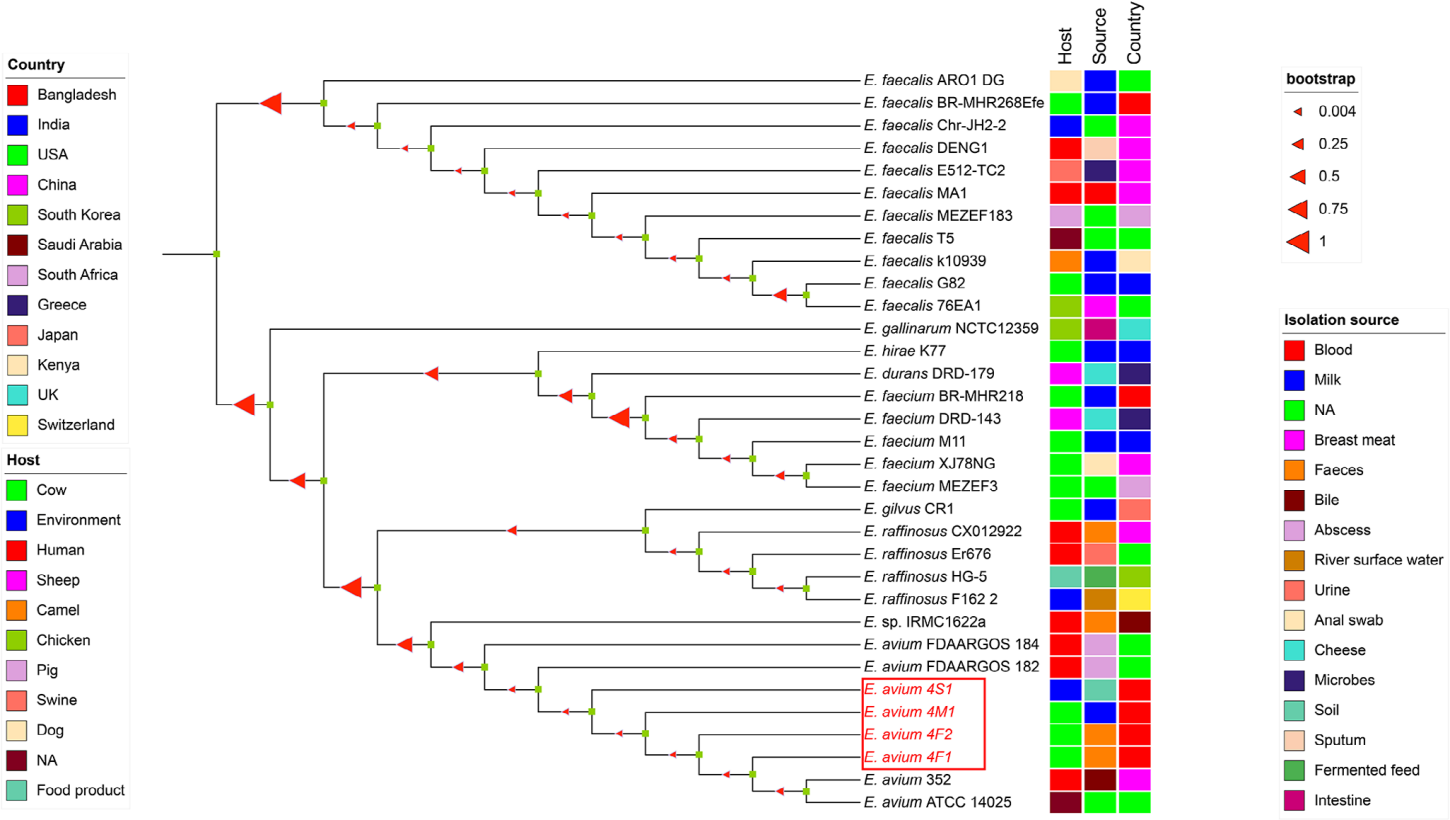
Maximum‐likelihood phylogeny of four *Enterococcus avium* strains from this study (4M1, 4S1, 4F1 and 4F2) and 29 reference *Enterococcus* genomes. Tip labels provide details on host, source and country. The four study strains are highlighted in the red box.

**FIGURE 6:**
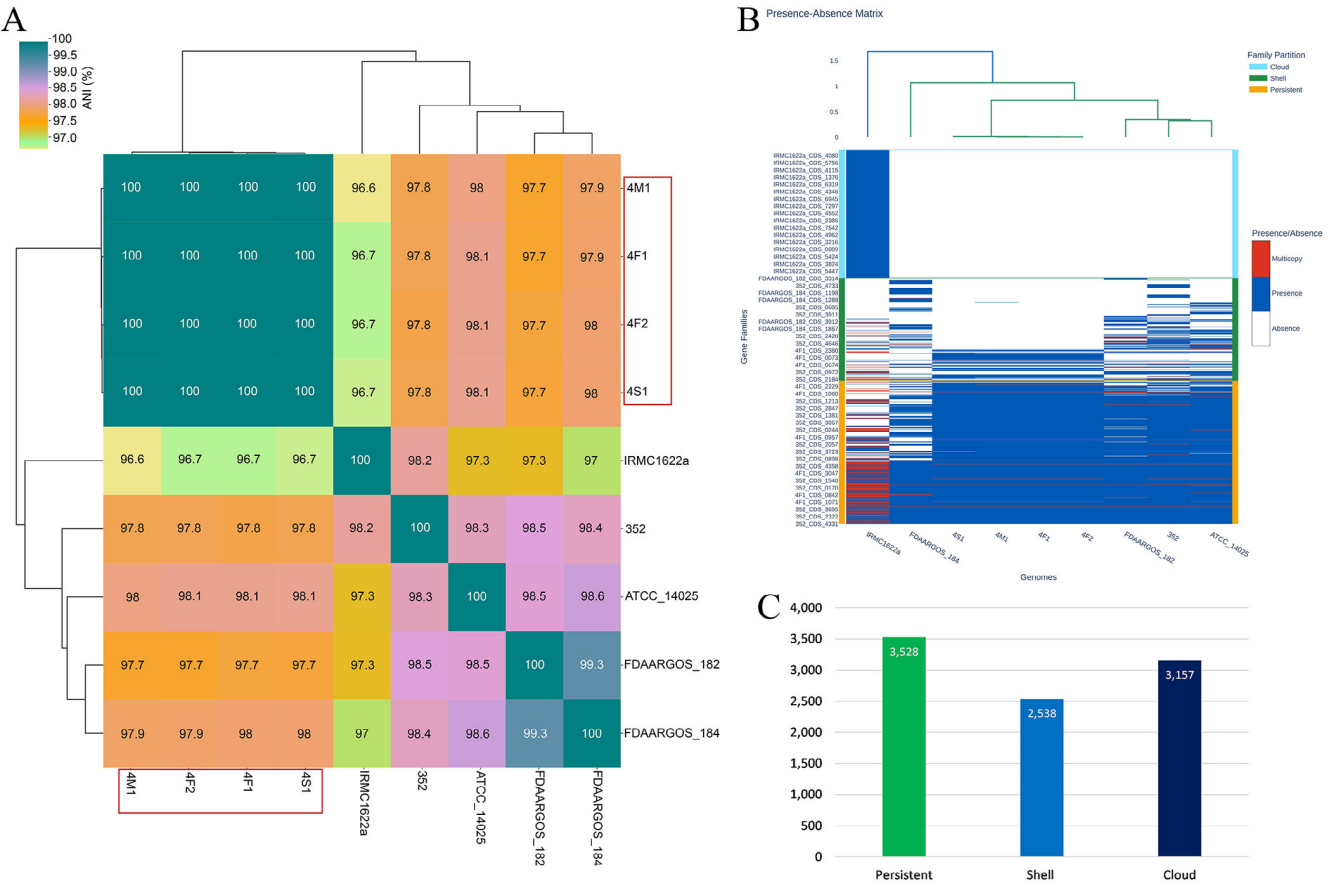
Comparative genomic analysis of four *Enterococcus avium* isolates (4M1, 4S1, 4F1 and 4F2) and five phylogenetically related *Enterococcus* genomes. (A) Clustered heatmap showing average nucleotide identity (ANI) values (%) among the genomes. The species‐level ANI threshold of 95% is indicated, with the study isolates highlighted by a red box. (B) Gene presence–absence matrix generated using PPanGGOLiN, illustrating the distribution of core, shell, cloud and multicopy gene clusters across the genomes. Each column represents a gene cluster, and each row represents a genome, with coloured blocks indicating gene presence. (C) Bar chart summarizing the number of persistent (core), shell and cloud gene families across the nine *Enterococcus* genomes.

A maximum likelihood phylogenetic analysis of the study genomes along with 29 reference genomes of *Enterococcus* spp. (Table S1) based on *16S rRNA* sequence revealed that all the draft genomes of 4M1, 4S1, 4F1 and 4F2 closely clustered along with *E. avium* ATCC 14025 (AHYV00000000), *E. avium* 352 (Yu et al. 2019), *E. avium* FDAARGOS_184 (CP024590.1), *E. avium* FDAARGOS_182 (NBSL00000000) and *Enterococcus* sp. IRMC1622a (JBAMHL000000000) genomes isolated from clinical sample from human (Figure 5). Further, ANI calculation of the phylogenetically related genomes revealed that there was more than 96% neucleotide identity between the genomes (Figure 6A). Notably, the study genomes had 100% nucleotide between them. Additionally, pangenome analysis of the *E. avium* genomes, conducted using PPanGGOLiN with the dataset used for ANI calculations, identified 3528 persistent gene families shared across all isolates, 2538 shell gene families variably present among strains and 3157 cloud gene families representing the most strain‐specific component (Figure 6B,C). A subset of multicopy gene families was also detected.

### 3.6 Functional Annotation Based on KEGG Pathways

**FIGURE 7:**
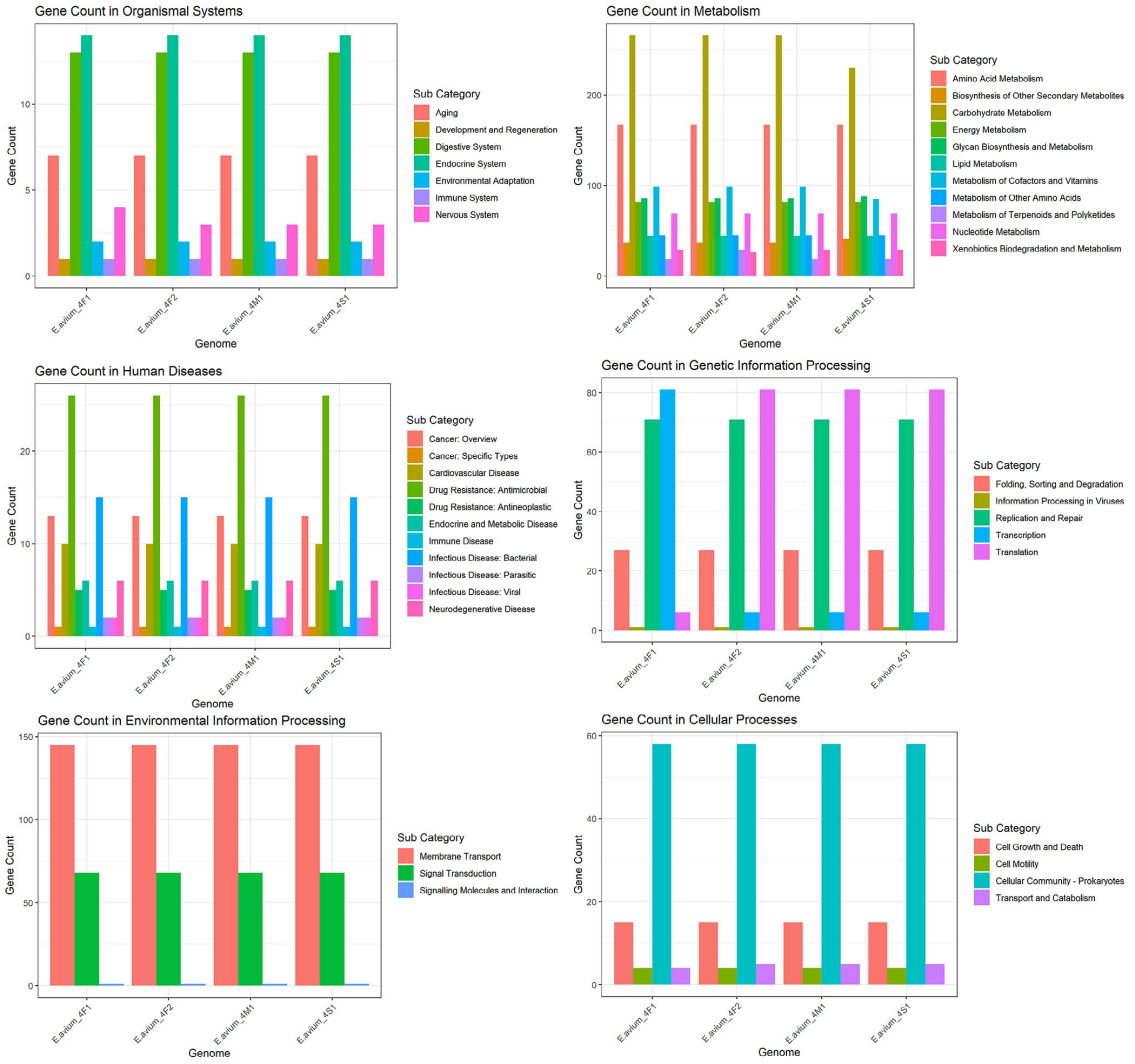
Metabolic functional potentials of *Enterococcus avium* strains 4M1, 4S1, 4F1 and 4F2 based on KEGG annotation. The figure presents detailed functional classes grouped into six main categories, with each category shown in a separate cluster for clarity.

The functional potential of the four *E. avium* genomes (4F1, 4F2, 4M1 and 4S1) was assessed through KEGG annotation, which assigned gene products to six major categories (Figure 7). Metabolism was the most represented category, with each genome encoding a large repertoire of genes involved in amino acid metabolism (167 genes), carbohydrate metabolism (266 genes in 4F1–4M1 and 230 in 4S1), glycan biosynthesis and metabolism (86–88 genes) and energy metabolism (82 genes). Within metabolism, pathways related to carbohydrate utilization and amino acid biosynthesis formed the dominant clusters, suggesting a strong genomic basis for nutrient acquisition and energy production. Genetic information processing accounted for a substantial proportion of the annotated genes, including replication and repair (71 genes), transcription (81 genes in 4F1; 6 genes in 4F2–4S1), translation (81 genes in 4F2–4S1; 6 genes in 4F1) and protein folding, sorting and degradation (27 genes). The variation in transcription‐ and translation‐related gene counts indicates subtle genomic differences among isolates that may influence regulatory capacity. Environmental information processing was also predominant, represented mainly by membrane transport (145 genes) and signal transduction (68 genes), highlighting the potential of *E. avium* to sense and respond to environmental cues. Cellular processes were comparatively less abundant, with genes related to cellular community–prokaryotes (58 genes) and cell growth and death (15 genes) being most frequent. Organismal systems comprised fewer genes overall, with consistent representation across isolates in pathways such as aging (7 genes), digestive system (13 genes) and the endocrine system (14 genes). Additionally, genes mapped to the human diseases category were also identified, including drug resistance–antimicrobial (26 genes), cardiovascular disease (10 genes) and infectious diseases: bacterial (15 genes) (Figure 7).

### 3.7 Bacteriocin Gene Clusters in *E. avium* Strains

Analysis of the four *E. avium* genomes (4F1, 4F2, 4M1 and 4S1) revealed the presence of multiple bacteriocin biosynthetic gene clusters (BGCs) (Figure S1A–P). All genomes contained gene cassettes encoding *Subtilosin_A*, *UviB* and *Zoocin_A*. The Subtilosin_A cluster consists of two core peptides, an associated ABC transporter/immunity protein and the biosynthetic protein AlbA (BmbF), which is characteristic of antilisterial bacteriocin production. In addition, the 4M1 and 4F1 genomes carried the *Bovicin_HJ50* gene cassette, the 4F2 genome harboured Thermophilin 1277 and the 4S1 genome contained Macedovicin.

### 3.8 Identification of ARGs in *E. avium* Strains

A total of six ARG‐associated features were identified in each *E. avium* strain (4S1, 4M1, 4F2 and 4F1), spanning three major resistance mechanisms: multidrug efflux systems, beta‐lactamase activity and fluoroquinolone resistance (File S1). All strains contained two genes encoding multidrug and toxic compound extrusion (MATE) family efflux pumps, accounting for 33.3% of the total ARG‐associated features in each genome. In addition, a single gene associated with PmrA‐family efflux‐mediated resistance was identified across all isolates. For beta‐lactam resistance, two distinct genes were found in each genome, one encoding a Class C beta‐lactamase and the other a metal‐dependent hydrolase associated with the beta‐lactamase superfamily. In addition, one gene each encoding DNA gyrase subunit A and subunit B, key targets of fluoroquinolone antibiotics, was also detected in all four genomes. Notably, minor variations were observed among strains, strain 4S1 and 4M1 each possessed complete fluoroquinolone resistance gene loci (both *gyrA* and *gyrB*), whereas 4F1 and 4F2 primarily showed single subunit representations (File S1). However, screening against comprehensive plasmid and resistance gene database (e.g., PlasmidFinder) revealed no detectable plasmid replicons or plasmid‐mediated resistance genes across all assembled contigs.

### 3.9 Virulence Gene Profiles Among *E. avium* Strains

**FIGURE 8:**
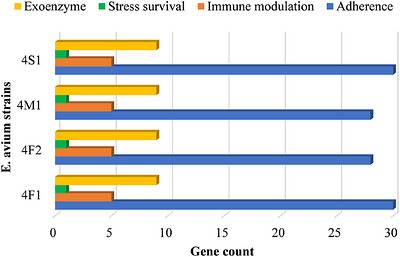
Distribution of virulence factor genes (VFGs) across *Enterococcus avium* strains 4M1, 4F1, 4F2 and 4S1. VFGs are classified into four functional categories: exoenzyme (yellow), stress survival (green), immune modulation (orange) and adherence (blue).

A total of 43–45 virulence genes were detected across the four *E. avium* strains (File S2, Figure 8). The predominant virulence category was adherence‐related genes, comprising over 70% of all detected factors. The *plr/gapA* gene encoding glyceraldehyde‐3‐phosphate dehydrogenase was the most abundant (*n* = 14 per strain), followed by *eno* (*n* = 9) and *ecbA/fss3* (*n* = 8). Adhesion‐related surface protein coding genes, such as *lap*, *tuf* and *sdrC/sdrF*, were also conserved across isolates, indicating a strong capacity for host colonization. Immune modulation genes (*rmlA, rfbA, rfbB, galU* and *SGO_RS05010*) were uniformly present, suggesting conserved mechanisms for immune evasion. The bile salt hydrolase (*bsh*) gene, associated with environmental persistence and gut survival, was identified in all strains. Minor variation was observed in *sdrC* and *sdrF* genes, which appeared only in isolates from faeces (4F1, 4F2) and soil (4S1) (File S2, Figure 8).

## 4 Discussion

Mastitis continues to impose substantial health and economic burdens on the dairy industry, where its aetiology now includes an expanding spectrum of emerging bacterial pathogens. The parallel rise of AMR among these agents compounds disease control challenges and elevates risks to both animal and human health. Dairy farm environments, as dynamic agro‐ecosystems, facilitate microbial exchange among livestock, humans and the surrounding environment, promoting the persistence and dissemination of resistance determinants. In this study, we conducted integrated phenotypic and genomic profiling of *E. avium*, an emerging mastitis‐associated pathogen, to characterize its resistance profile, virulence repertoire and adaptive genomic traits, providing insights for targeted mitigation strategies and highlighting its potential public health significance. Antibiogram analysis of *E. avium* isolates highlights their high prevalence of multidrug resistance, reflecting an adaptive capacity in dairy farm environments that compromises the effectiveness of commonly used antimicrobials. Resistance to key frontline antibiotics, including sulphonamides, oxacillin, doxycycline and tetracycline, underscores the challenges of empirical treatment, whereas retained susceptibility to gentamicin points to its continued therapeutic potential. The emergence of extreme MDR phenotypes reflects selective pressures from veterinary antibiotic use and emphasizes the need for ongoing AMR surveillance and the development of alternative treatment strategies.

Comprehensive genome analysis provides profound insights into genetic composition, functional potential and adaptive traits, thereby advancing understanding of microbial diversity, pathogenicity and evolutionary dynamics (Mallick et al. 2025; Allen and Banfield 2005). Whole‐genome analysis of four MDR *E. avium* isolates from a dairy farm, including milk (4M1), faeces (4F1 and 4F2) and soil (4S1), demonstrates a highly conserved genomic structure, reflecting high genomic similarity among the studied isolates across diverse environmental niches. Phylogenetic and ANI analyses indicate that dairy farm *E. avium* isolates are highly conserved, forming a single clade with complete nucleotide identity. Their close clustering with human‐derived *Enterococcus* strains suggests possible ecological relatedness and underscores the ecological versatility of *E. avium*, warranting further investigation into its public health relevance. This evolutionary stability is further supported by the persistent genome, which underpins essential housekeeping and metabolic pathways (Kweon et al. 2015). Pangenome analysis revealed a conserved core of persistent gene families alongside a substantial accessory genome of shell and cloud genes, indicating genomic variability and strain‐specific functions. The detection of multicopy gene families suggests functional redundancy and adaptive potential, reinforcing the capacity of *E. avium* to persist and adapt across diverse ecological niches.

In KEGG annotation of the study *E. avium* genomes highlights a strong metabolic capacity, particularly in carbohydrate and amino acid metabolism, suggesting adaptability for nutrient acquisition and energy production (Huang et al. 2025). Variation in transcription‐ and translation‐related genes indicates subtle differences in regulatory potential among isolates. Prominent membrane transport and signal transduction genes reflect the ability to sense and respond to environmental cues (Xie et al. 2025). Notably, the presence of AMR and human disease–associated pathways aligns with observed multidrug resistance and suggests the presence of virulence‐associated traits, although their functional relevance requires further validation, highlighting the clinical relevance of these isolates. Bacteriocins are ribosomally synthesized antimicrobial peptides produced by bacteria that can inhibit the growth of closely related or competing pathogenic strains, contributing to microbial competition, niche colonization and potential modulation of pathogenicity (Micenková et al. 2014; Snopkova et al. 2020).

The identification of multiple bacteriocin BGCs in *E. avium* strains 4F1, 4F2, 4M1 and 4S1 highlights their potential role as competitors within the bovine mastitis microbiome. All four strains harbour a gene cassette for *subtilosin A*, a Class IIa bacteriocin with broad‐spectrum activity against Gram‐positive pathogens (Carson et al. 2017). Additional BGCs include *Zoocin_A*, *UviB*, Macedovicin, Thermophilin 1277 and a Bovicin HJ50‐like lantibiotic, each known for activity against pathogenic bacteria (Vidal Amaral et al. 2022; Siddique et al. 2025). These bacteriocins likely enhance microbial fitness by inhibiting competing microorganisms, facilitating colonization and persistence in the mammary gland. The expanded bacteriocin repertoire may indirectly support pathogenic success though ecological dominance within the mastitic environment (Sharp and Foster 2025). Further investigation is warranted to delineate the specific role of these bacteriocins in the pathogenesis of mastitis.

Importantly, the uniform detection of multidrug efflux systems, beta‐lactamases and fluoroquinolone resistance genes across *E. avium* strains underscores the intrinsic adaptability of this species in dairy farm environments. Efflux‐mediated resistance, particularly via MATE and *PmrA* families, is a conserved mechanism in enterococci, promoting extrusion of structurally diverse antimicrobials such as tetracycline, doxycycline and azithromycin, as well as toxic compounds, thereby enhancing survival under antimicrobial pressure (Poole 2007). The presence of Class C and metallo‐beta‐lactamases corresponds with resistance to β‐lactam antibiotics, including ampicillin and cephalosporins (Hollenbeck and Rice 2012), whereas the detection of *gyrA* and *gyrB* genes indicates potential fluoroquinolone resistance, including ciprofloxacin and nalidixic acid (Petersen and Jensen 2004; Campioni et al. 2017). The conservation of ARG profiles across milk, faecal and soil isolates suggests strong genomic stability or horizontal gene transfer, highlighting the role of enterococci in agricultural environments as reservoirs of clinically relevant resistance determinants. However, observed discrepancies between the phenotypic antimicrobial susceptibility and the genotypically predicted ARGs in *E. avium* may reflect differences in genome coverage and database annotation limitations, as well as additional resistance mechanisms such as chromosomal mutations, efflux‐mediated resistance, regulatory changes, inducible gene expression and the substrate specificity of encoded resistance proteins (Mahfouz et al. 2020; Koolman et al. 2025; Aiezza et al. 2023). Further, virulome analysis of the *E. avium* strains revealed high conservation across isolates from different sources, aligning with previous reports that enterococci maintain a stable core virulence repertoire (Bag et al. 2022; Fisher and Phillips 2009). The predominance of adhesion‐related genes such as *plr/gapA*, *eno* and *ecbA* reflects the importance of biofilm formation and tissue colonization (Kim et al. 2022; Rodrigues et al. 2022). The presence of *bsh* across all isolates indicates adaptive capacity to intestinal or environmental stress (Yu et al. 2019). The soil isolate (4S1) exhibited a slightly broader adhesin profile (*sdrC/sdrF*), possibly reflecting adaptation to environmental persistence. Notably, virulence gene composition of milk isolate 4M1 closely resembled that of faecal isolates, suggesting possible ecological association between isolates from different sources within the farm environment. Collectively, this study underscores the genomic stability, multidrug‐resistance and virulence potential of *E. avium* strains present in dairy environments, highlighting their relevance to both animal and public health. However, the limited sample size and the focus on a single dairy farm restrict the generalizability and broader applicability of the findings. Furthermore, the lack of mutation‐based and experimental validation analyses hinders a definitive understanding of genotype–phenotype relationships and limits confidence in inferring transmission dynamics. Future research incorporating broader geographic sampling and functional validation of key genes is necessary to fully elucidate the ecological and pathogenic roles of this emerging mastitis‐associated pathogen.

## 5 Conclusion

This study indicates that *E. avium* is an emerging MDR bacterium in dairy farm environments, with high phenotypic resistance and conserved genomic features across milk, faecal and soil isolates. Genomic analysis revealed a repertoire of ARGs, virulence‐associated determinants, bacteriocins and metabolic pathways, suggesting ecological adaptability and possible persistence in mastitis‐associated niches. Although phylogenetic similarity with human‐derived enterococci was observed, its public health relevance remains putative and requires further investigation. The detection of efflux pumps, β‐lactamases and fluoroquinolone‐associated genes highlights dairy environments as potential reservoirs of AMR determinants. Overall, these findings emphasize the ecological persistence and resistance potential of *E. avium* in dairy systems and support the need for continued One Health–oriented surveillance and further studies on its transmission dynamics and pathogenic mechanisms.

## Author Contributions

M. Nazmul Hoque conceived and designed the study. Monira Rahaman, Md. Morshedur Rahman, Kh. Yeashir Arafat, Md. Abu Ahsan Gilman, Naim Siddique and Taniya Sultana conducted the experiments, curated and analysed the data, performed visualization and prepared the original draft. Ziban Chandra Das, Anup Kumar Talukder, Tofazzal Islam and A. N. M. Aminoor Rahman critically reviewed and revised the manuscript. All authors read and approved the final version of the manuscript.

## Funding

This research was supported by a grant from the Bangladesh Bureau of Educational Information and Statistics (BANBEIS), Ministry of Education, Government of the People's Republic of Bangladesh (Grant no. LS20221764, duration 2023–2025).

## Ethics Statement

This study was reviewed and approved by the Animal Research Ethics Committee (AREC) of the Gazipur Agricultural University, Bangladesh (Reference number: FVMAS/AREC/2023/6679, Date: 16/01/2023).

## Conflicts of Interest

The authors declare no conflicts of interest.

## Supporting information




**Supporting Material 1**: vms371060‐sup‐0001‐SuppMat.docx

**Supporting Material 2**:vms371060‐sup‐0002‐FigureS1.jpg

**Supporting Material 3**:vms371060‐sup‐0003‐SuppMat.xlsx

**Supporting Material 4**:vms371060‐sup‐0004‐SuppMat.xlsx

## Data Availability

The *16S rRNA* gene sequences of *E. avium* strains 4M1, 4S1, 4F1 and 4F2 have been deposited in NCBI GenBank under accession numbers PX487938, PX487939, PX487936 and PX487937, respectively. The whole genome sequences for these strains are available in GenBank and the NCBI Sequence Read Archive (SRA) under BioProject accessions PRJNA1046255. The genome versions reported in this study are JAXHVX000000000 for 4M1, JAXHVY000000000 for 4S1, JAXHVW000000000 for 4F1 and JAXHVV000000000 for. All datasets generated for this study are included in this article and the Supporting Information files.
