# Supplementary material for: Genomic Insights Into Antimicrobial Resistance and Virulence of Enterococcus avium Strains From Bovine Mastitis in Some Selected Dairy Farms of Bangladesh
Source: Vet Med Sci. 2026 Jun 29;12(4):e71060. doi: 10.1002/vms3.71060 (PMC13312985; doi:10.1002/vms3.71060)
Supplement: Supplementary file 1 — Supporting Material 1: vms371060‐sup‐0001‐SuppMat.docx [file VMS3-12-e71060-s001.docx]

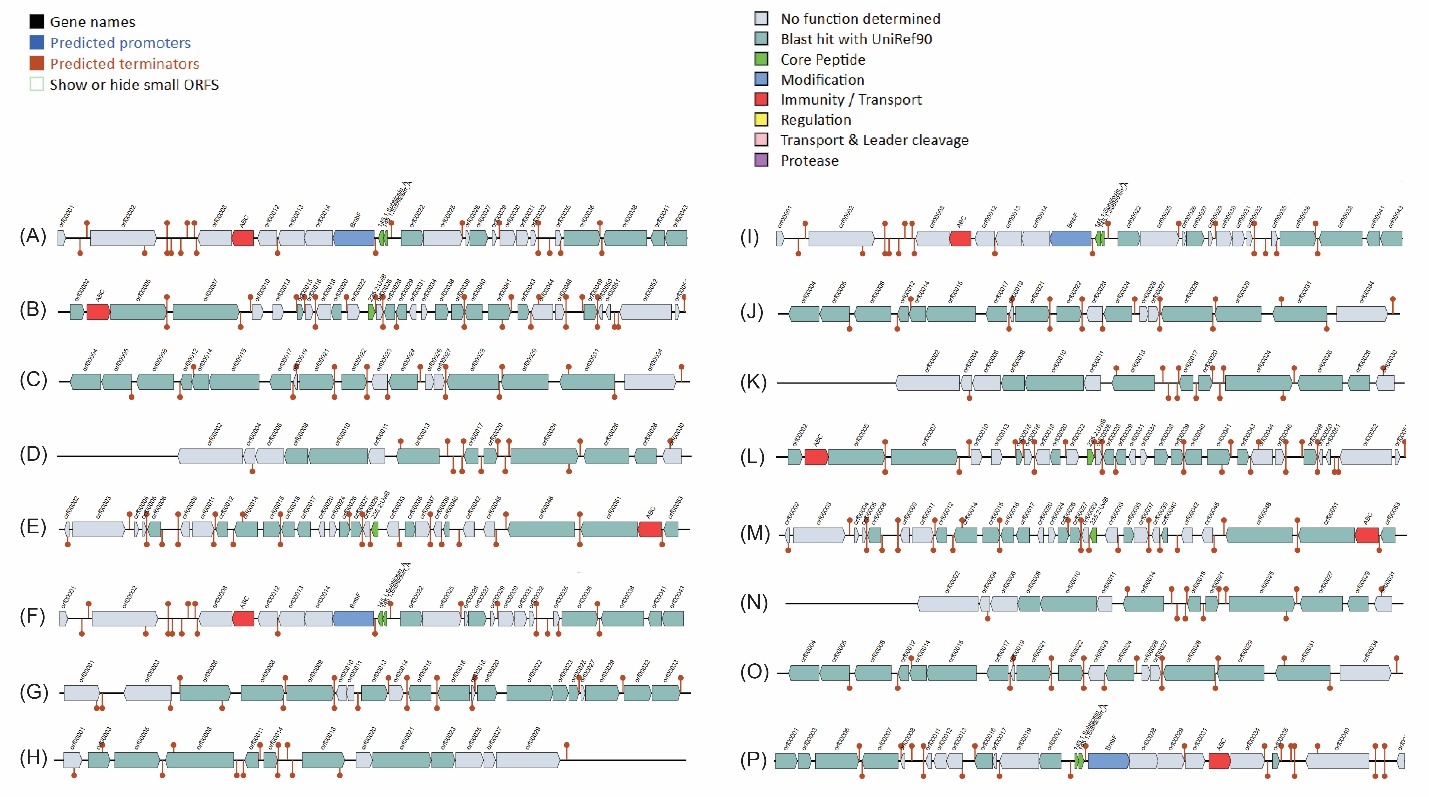


**Figure S1.** Distribution and diversity of bacteriocin gene cassettes in *Enterococcus avium* strains 4M1, 4F1, 4F2, and 4S1. In strain 4M1, the bacteriocins are **(A)** Subtilosin_A, **(B)** UviB, **(C)** Bovicin_HJ50, and **(D)** Zoocin_A; in 4F1, **(E)** UviB, **(F)** Subtilosin_A, **(G)** Bovicin_HJ50, and **(H)** Zoocin_A; in 4F2, **(I)** Subtilosin_A, **(J)** Thermophilin1277, **(K)** Zoocin_A, and **(L)** UviB; and in 4S1, **(M)** UviB, **(N)** Zoocin_A, **(O)** Macedovicin, and **(P)** Subtilosin_A.

**Table S1.** Genome accession ID, isolation source, host, disease, and country of origin of 33 *Enterococcus* genomes

| **Name** | **Accession** | **Source** | **Host** | **Disease** | **Country** |
| --- | --- | --- | --- | --- | --- |
| *Enterococcus faecalis* k10939 | JACTAL000000000 | Milk | Camel | Mastitis | Kenya |
| *Enterococcus faecalis* 76EA1 | LEQG00000000 | Breast meat | Chicken | NA | USA |
| *Enterococcus avium* ATCC_14025 | AHYV00000000 | NA | NA | NA | USA |
| *Enterococcus avium* _352 | NZ_CP034169 | Bile | Human | Colelithiasis | China |
| *Enterococcus gallinarum* NCTC12359 | UFYU00000000 | intestine | Chicken | NA | UK |
| *Enterococcus faecium* MEZEF3 | JAHHFJ000000000 | NA | Cow | NA | South Africa |
| *Enterococcus faecalis* MEZEF183 | JAHHEQ000000000 | NA | Pig | NA | South Africa |
| *Enterococcus faecalis* T5 | ASDH00000000 | NA | NA | NA | USA |
| *Enterococcus avium* FDAARGOS_184 | CP024590.1 | Abscess | Human | Abscess | USA |
| *Enterococcus raffinosus*_CX012922 | CP081846.1 | feces | Human | Crohn | China |
| *Enterococcus raffinosus* F162_2 | CP072888.1 | River surface water | Environment | NA | Switzerland |
| *Enterococcus faecalis* DENG1 | CP004081 | Sputum | Human | Pneumonia | China |
| *Enterococcus faecalis* MA1 | ANMP00000000 | Blood | Human | Cholangitis | China |
| *Enterococcus raffinosus* Er676 | CP104764.1 | Urine | Human | Chronic Cystitis | USA |
| *Enterococcus raffinosus* HG-5 | CP104392.1 | fermented food | Food Product | NA | South Korea |
| *Enterococcus gilvus* CR1 | CP030932.1 | Milk | Cow | NA | Japan |
| *Enterococcus faecalis* Chr-JH2-2 | CP100596.1 | NA | Environment | NA | China |
| *Enterococcus faecalis* E512-TC2 | CP086566.1 | Microbes | Swine | NA | China |
| *Enterococcus faecium* XJ78NG | CP109758.1 | Anal Swab | Cattle | NA | China |
| *Enterococcus durans* DRD-179 | JAMRUQ000000000 | Cheese | Sheep | Healthy | Greece |
| *Enterococcus faecium* DRD-143 | JAMRUZ000000000 | Cheese | Sheep | Healthy | Greece |
| *Enterococcus avium* FDAARGOS_182 | NBSL00000000 | Abscess | Human | Abscess | USA |
| *Enterococcus faecalis* BR-MHR268Efe | JAOBXK000000000 | Milk | Cow | Mastitis | Bangladesh |
| *Enterococcus faecium* BR-MHR218 | JAODSZ000000000 | Milk | Cow | Mastitis | Bangladesh |
| *Enterococcus sp.* IRMC1622a | JBAMHL000000000 | Feces | Human | Gastrointestinal infection | Saudi Arabia |
| *Enterococcus avium* 4S1 | JAXHVY000000000 | Soil | Environment | Mastitis | Bangladesh |
| *Enterococcus avium* 4M1 | JAXHVX000000000 | Milk | Cow | Mastitis | Bangladesh |
| *Enterococcus avium* 4F2 | JAXHVV000000000 | Feces | Cow | Mastitis | Bangladesh |
| *Enterococcus avium* 4F1 | JAXHVW000000000 | Feces | Cow | Mastitis | Bangladesh |
| *Enterococcus faecalisI* G82 | JBEUZZ000000000 | Milk | Cow | Mastitis | India |
| *Enterococcus faecalis* ARO1_DG | ACAK00000000 | Milk | Dog | Mastitis | USA |
| *Enterococcus faecium* M11 | JBEUZQ000000000 | Milk | Cow | Mastitis | India |
| *Enterococcus hirae* K77 | JBEUZI000000000 | Milk | Cow | Mastitis | India |
