# Supplementary figures and images for: Genomic Insights Into Antimicrobial Resistance and Virulence of Enterococcus avium Strains From Bovine Mastitis in Some Selected Dairy Farms of Bangladesh
Source: Vet Med Sci. 2026 Jun 29;12(4):e71060. doi: 10.1002/vms3.71060 (PMC13312985; doi:10.1002/vms3.71060)

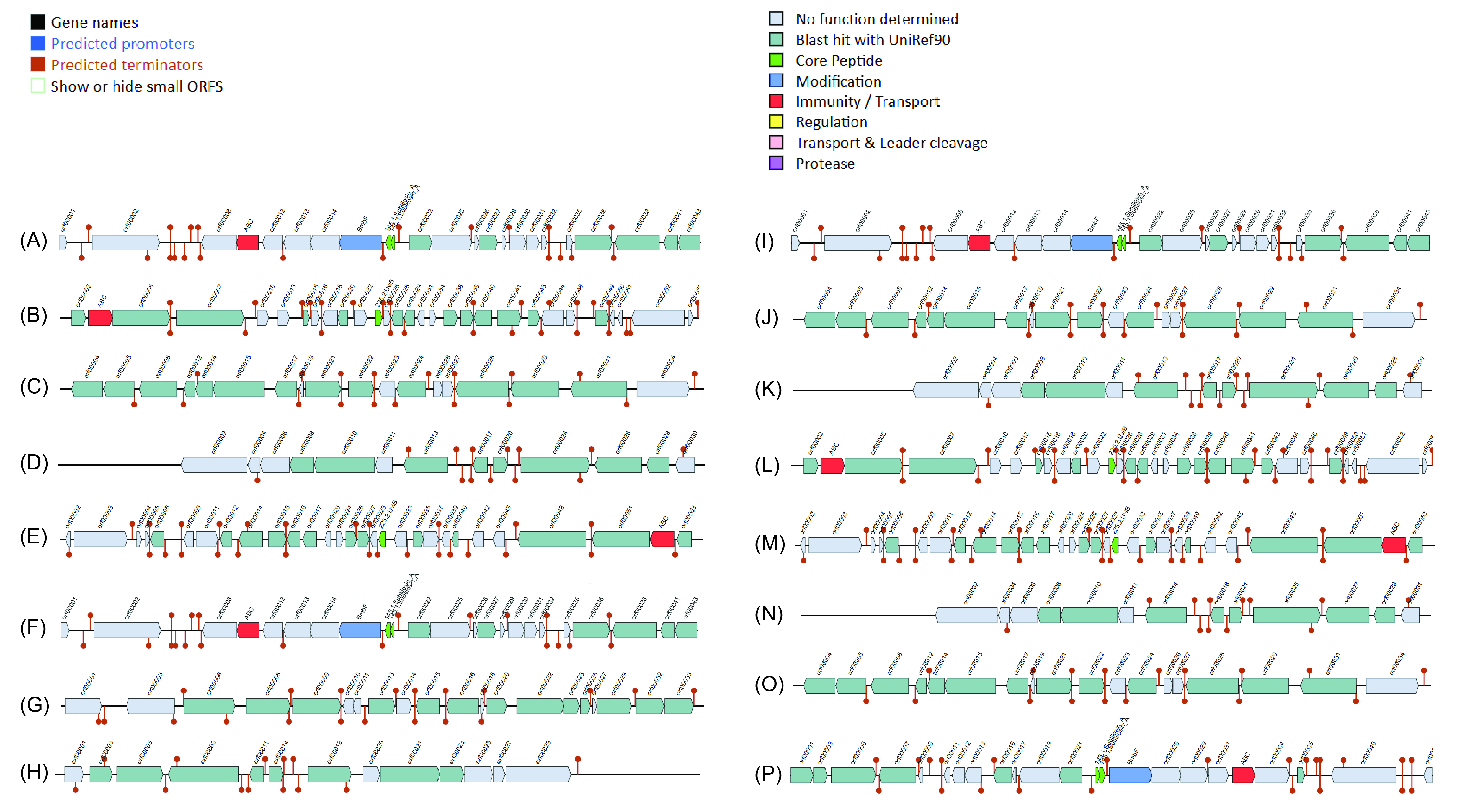

Supplement: Supplementary file 2 — Supporting Material 2:vms371060‐sup‐0002‐FigureS1.jpg [file VMS3-12-e71060-s003.jpg]
